# The Xiangya Ocular Tumor Bank: A Disease-Specific Biobank for Advancing Translational Research Into Ocular Tumors

**DOI:** 10.3389/fmed.2021.774624

**Published:** 2022-01-27

**Authors:** Zhaolin Gao, Jia Tan, Sha Wang, Haiyang Yu, Ziyu Zhou, Yun Zhang, Mushi Zhou, Xiaobo Xia, Fei Yao, Jufang Huang

**Affiliations:** ^1^Department of Anatomy and Neurobiology, School of Basic Medical Science, Central South University, Changsha, China; ^2^Department of Ophthalmology, Xiangya Hospital, Central South University, Changsha, China; ^3^Hunan Key Laboratory of Ophthalmology, Central South University, Changsha, China

**Keywords:** ocular tumor, biospecimen, clinical data, translational research, biobank

## Abstract

The pathogenesis and etiology of various ocular tumors remain largely unclear, limiting the development of diagnostic and treatment approaches for such tumors. Tissue samples from patients are also valuable resource to elucidate mechanisms underlying tumorigenesis. Here we present the early phase setup of an ocular tumor biobank at Xiangya Hospital. Blood and tissue samples along with associated clinical data were obtained from patients who underwent surgery in the Department of Ophthalmology of Xiangya Hospital from December 1, 2018 to January 31, 2020. Standardized operating protocols were developed for the collection, transportation, processing and preservation of ocular tumor samples. A total of 92 clinical cases suffered from 21 types of eye tumors and several undiagnosed eye diseases were covered. A total of 846 samples were preserved in the ocular tumor biobank, including 356 blood samples (42.1%), 324 plasma samples (38.3%), and 166 tissue samples (19.6%). Using the clinical data, we analyzed the prevalence of malignant ocular tumors in association with variables of age, gender, tumors' location and size, and presenting complaints of lump and proptosis. The factors predictive of malignant ocular tumors, included gender (B = 1.599; *P* = 0.025) and the symptom of proptosis (B = −2.534; *P* = 0.001). Overall, the setup of clinically-based ophthalmologic biobank could support pathological and translational research into ocular tumors.

## Introduction

The ocular tumor is a kind of life-threatening disease that may cause vision loss as well as other disabilities. A tumor in the eye can originate from any eye tissue, resulting in melanocytic, lymphoid, leukemic, fibrous, epithelial, lipomatous, and other forms of lesions (1). Retinoblastoma is the most common primary intraocular malignancy of childhood. In the United States and Northern Europe, the mean incidence of retinoblastoma is around 11 new cases per million individuals under 5 years old. It appears to uniformly occur across populations, ranging from one in 16,000–18,000 live births (2, 3). Based on worldwide epidemiological data, annually between 7,202 and 8,102 children suffer from retinoblastoma (4). Uveal melanoma is the most common primary intraocular malignancy in adults, with the incidence in the United States has remained stable at ~5.1 per million since 1973 (5). Since it can metastasize at an early stage and resist treatment when metastasis occurs, the long-term mortality of uveal melanoma exceeds 50% (6, 7). Although the incidence of most ocular tumors is generally not high, the consequences are serious, causing loss of vision and even threatening lives.

Several animal and cell culture models have contributed significantly to our current understanding of the biological mechanisms underlying ocular tumors, but these approaches have their limitations. Cell culture models for these tumors are scarce, while eye tumors are complex and pathologically diverse. Long term cell line cultivation has a limitation regarding the maintenance of the crucial driver gene expression or the tumors' growth behavior. For instance, Y79 is a commonly used human retinoblastoma cell line, but it undergoes significant changes and/or selection in cell culture that distinguish it from the primary human retinoblastomas (8). For *in vivo* systems, developing suitable animal models that recapitulate ocular tumors' structural and functional properties remains difficult. For instance, a study of a mouse model injected with UMT2 cells presented a high degree of similarity between the tumors generated in the mouse eyes and the corresponding primary human uveal melanoma, but without mimicking its metastatic property (9). The epigenetic landscape of human retinoblastoma differ significantly from mouse counterpart. Some candidate pathways for targeted molecular therapies of human retinoblastoma are not deregulated in mouse tumors (10). All the above greatly hampers the progress of basic research and therapeutic advances for ocular tumors.

For the development of novel diagnosis and treatment of ocular tumors, human-derived samples are optimal to study the disease mechanism. Specifically, human-derived biospecimens maintain a greater genetic heterogeneity of native tumor tissue relative to animal samples. To date, human samples of eye tumors remain rarely available. At least in China, the awareness for the importance of biobanking appears to be much limited among ophthalmologists as well as patients, owing to limited knowledge about biobanking or concerns about the privacy issue. On the other hand, an online survey by the Association for Research in Vision and Ophthalmology (ARVO) reported that 43% of its members are challenged to obtain an adequate quantity of human eye tissue for their research (11). Biobanks are biorepositories that accept, process, store, and distribute biospecimens and/or associated data for research and clinical care (12), which can provide a potential strategy for alleviating the shortage of eye tumor resources. There are several eye tissue biobanks established in the world, including the US NIH's National Eye Institute which supports the NEIBank that provides eye tissues and derivatives for research, and the University of Liverpool, Ocular Oncology Biobank (http://www.loorg.org/disease-focus-and-research.html). Given the lack of such biobanks in China, we are building an ocular tumor biobank to facilitate translational and molecular studies toward better clinical diagnosis and treatment of eye tumors. In this report, we describe the operational protocols for the collection of biosamples and clinical dataset. We also extend some preliminary results based on the characterization of the banked materials.

## Materials and Methods

### Aims and Design

The current work's primary aims were to describe the setup of our disease-specific biobank and the characterization of the ocular tumor samples. We also present some preliminary results based on the overview of the collection of biological specimens and associated clinical data. We also seek to provide guidance on how to develop and operate a successful biobank. Further, we attempt to assist clinicians with eye tumor diagnosis and treatment by analyzing the available ocular biosamples relative to corresponding clinical data.

**Figure 1:**
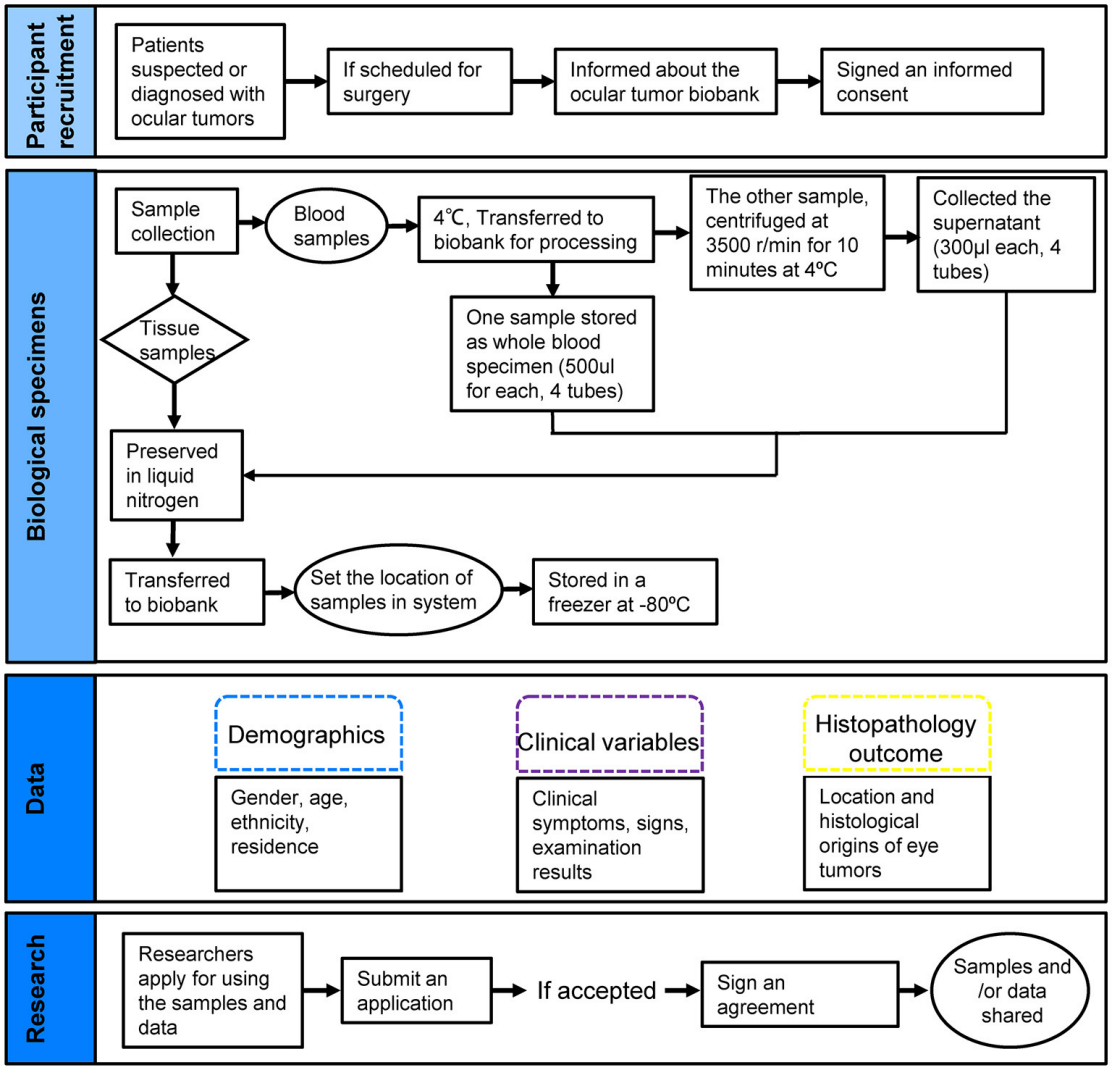
Presentation of the ocular tumor biobank workflow.

The Xiangya ocular tumor bank, mainly supported by the National Key R&D Program of China and based on collaboration with the Department of Ophthalmology of Xiangya Hospital Central South University, is a dedicated biobank for ocular tumors. The biobank holds biological materials and associated clinical data from patients with ocular tumors, including epidemiological, imaging, tumor characteristics, and follow-up data. Ophthalmologists were responsible for signing an informed consent with patients to include their biometric and clinical data as well as eye tumor samples whenever available at the time of surgery. We oversaw the follow-up work, including transportation, processing, and preservation of samples and data storage with specialized software used for recording of all biobanking events. Only authorized persons can enter the platform. Applications should be submitted to the scientific board for researchers who want to access biospecimens and/or data. If the request is accepted, agreements are signed between the research institutions under the laws and regulations in force, and the data and/or samples are made available (Figure 1).

### Recruitment of Participants

All patients who entered the Xiangya Hospital Central South University to evaluate suspected or known ocular tumors, and are scheduled for surgery, will be informed about the ocular tumor biobank and asked if they are willing to participate. If patients agree, they will be asked to sign an informed consent to include their data and samples in the biobank. The ocular tumor biobank has been approved by the institutional review board (IRB) of the Third Xiangya Hospital of Central South University (2016-S147). The biobank collected samples from the participants who underwent surgery in Department of Ophthalmology of Xiangya Hospital from December 1, 2018 to January 31, 2020. Blood and tissue samples and associated clinical data of electronic medical records were systematically collected.

### Collection, Transportation, Processing, and Preservation of Samples

We first confirmed with patients' ophthalmologists before each surgery about collecting samples. Information for each patient was entered into the biobank management system in advance and a unique barcode label for each sample was created. Tubes were prepared for sample collection and delivered to physicians responsible for performing surgery and collecting samples. Blood and tumor tissue were routinely collected during surgery. Other types of samples, such as bone marrow and cerebrospinal fluid, are also valuable resources for the study of ocular tumors, which could be available in case that the patients needed necessary invasive examinations. However, we have not considered it mandatory to collect such samples for obvious ethical reasons. The procedures for biological material collection and storage were developed following the best practice guidelines issued by the International Society for Biological and Environmental Repositories (ISBER) (13). Blood was collected into two EDTA tubes (6 ml). One tube was used to prepare the whole-blood sample and the other tube was centrifuged for preparing plasma aliquots. The ocular tumor sample collection followed the general principle of established guidelines (13). We ensured that the samples were clean and moist. Associated data including the basic information of donors and samples was recorded. The sample was collected as the following. Enucleations performed in patients with retinoblastoma or choroidal melanoma were similar to operational techniques described by Lang et al. (14). After the patient was anesthetized, a 360° conjunctival periotomy was completed and the Tenon's layer was dissected away from the sclera. All four rectus muscles were sequentially isolated with muscle hooks, secured with 5–0 sutures and transected from the sclera. Using traction on the medial rectus insertion site, the optic nerve was palpated and transected in the posterior orbit using long-tipped scissors. The isolated eyeball was put onto surgical dressing soaked in saline. Cutting the corneal tissue along the limbus, a radial incision of sclera was made from each corneal incision to the optic disc. Then we turned the cut sclera up to expose the tumor tissue in the eyeball. For patients with retinoblastoma, an opaque and white dome-shaped retinal mass arises from the inner retinal layers extending toward the vitreous cavity. We collected and preserved 0.2 × 0.2 × 0.2 cm tumor tissue as samples, avoiding taking the necrosis and liquefaction components of the tumor. The remaining eyeball was sent for pathological examination. For the patients with choroidal melanoma, the tumor appeared as a mass deep inside the retina. We could see the dome-shaped or mushroom-shaped retinal mass that extended toward the vitreous cavity with part of the retina covering the tumor. We separated the retina from tumor carefully. We collected the same size of tissue we took from retinoblastoma tumors and preserved it, avoiding the necrosis and liquefaction components of the tumor. The remaining eyeballs were sent for pathological examination.

Following collection, tissue samples were deposited into liquid nitrogen immediately. In contrast, blood samples were stored in a container at 4°C and then transferred to the laboratory for further processing, with a maximum delay of 30 min. One blood sample was transferred into 4 × 1.5 mL cryogenic aliquot tubes for storage of the whole blood sample (500 μl each). The other blood sample was centrifuged at 3,500 r/min for 10 min at 4°C and the supernatant was transferred into 4 ×1.5 ml cryogenic aliquot tubes (300 μl each). All aliquots were labeled with a unique corresponding barcode that included subject ID, diagnosis, collection date, and biospecimen type. Following the above processing, all specimens were preserved in liquid nitrogen and transported to the eye tumor biobank. The aliquots were stored in a locked freezer at −80°C until future use. Location of aliquots should be entered into the biobank management platform in advance. The storage temperature was monitored through continuous recordings, and the freezers were connected to a central alarm system.

### Clinical Data Collection

In addition to specimens, associated clinical data retrieved from the hospital's electronic health care system was collected. Data pertains to demographics, clinical features, and histopathology outcomes. Demographic data includes gender, age, ethnicity, and the address of each participant was collected. Clinical variables collected were described in terms of main symptoms, duration of symptoms, visual acuity, intraocular pressure, tumor size, tumor location, lymph node metastasis, eye movement, as well as the family history of cancer and any follow-up. The details of smoking and drinking habits were also recorded. Histopathology variables concerning histological type, together with tumor differentiation, were obtained from the pathology department of the hospital. We kept the results in our system linked to the corresponding samples in the biobank. No additional immunohistochemistry associated with the diagnosis of the tumors was performed. We extracted each participant's information from the electronic health care system to better understand event rates and to aid in future study design.

### The Application of Specialized Software for Biobank Management

The software we used is a specialized software system which has comprehensive functions for governing the biobank. The software's main functions involve two parts, tracing the location of incoming and outgoing samples as well as recording the detailed information of donors and samples. The administrator of the biobank who was authorized to enter the system was given a unique login identification. When he stored the samples in the biobank, he chose which refrigerator and where in the refrigerator to store the samples in the system. Then he put samples in the corresponding location of the refrigerator. When a researcher applied to access the samples, the administrator recorded the outgoing information of the samples in the system and the samples could be retrieved after receiving permission from the governance committee. We also used the software to record the donors' information such as gender, age, ethnicity, medical record number, examination results and diagnosis. Information including sample types, volume, collection time, and number of freeze-thaw cycles was also recorded. Any other associated data that we want to record can be added to the software. Furthermore, the information recorded in the software can be downloaded in the form of spreadsheets and used for further analyses.

### Statistical Analysis

Information concerning biological samples and related clinical data were included for analysis. All statistical analyses were performed using IBM SPSS 22.0. Continuous data were characterized as the mean and range. An independent samples *t*-test was used to compare the continuous variables between both groups. Categorical data in each group were presented as numbers and percentages and compared between the groups using the Chi-square test. The Fisher exact test was applied for those cases in which the expected frequencies were <5. A multivariate logistic regression model was used to identify the predictive risk or protective factors of eye tumors. For all tests, probability values of *p* < 0.05 were reported as statistically significant.

## Results

### Overview of Specimen Collection

**Figure 2:**
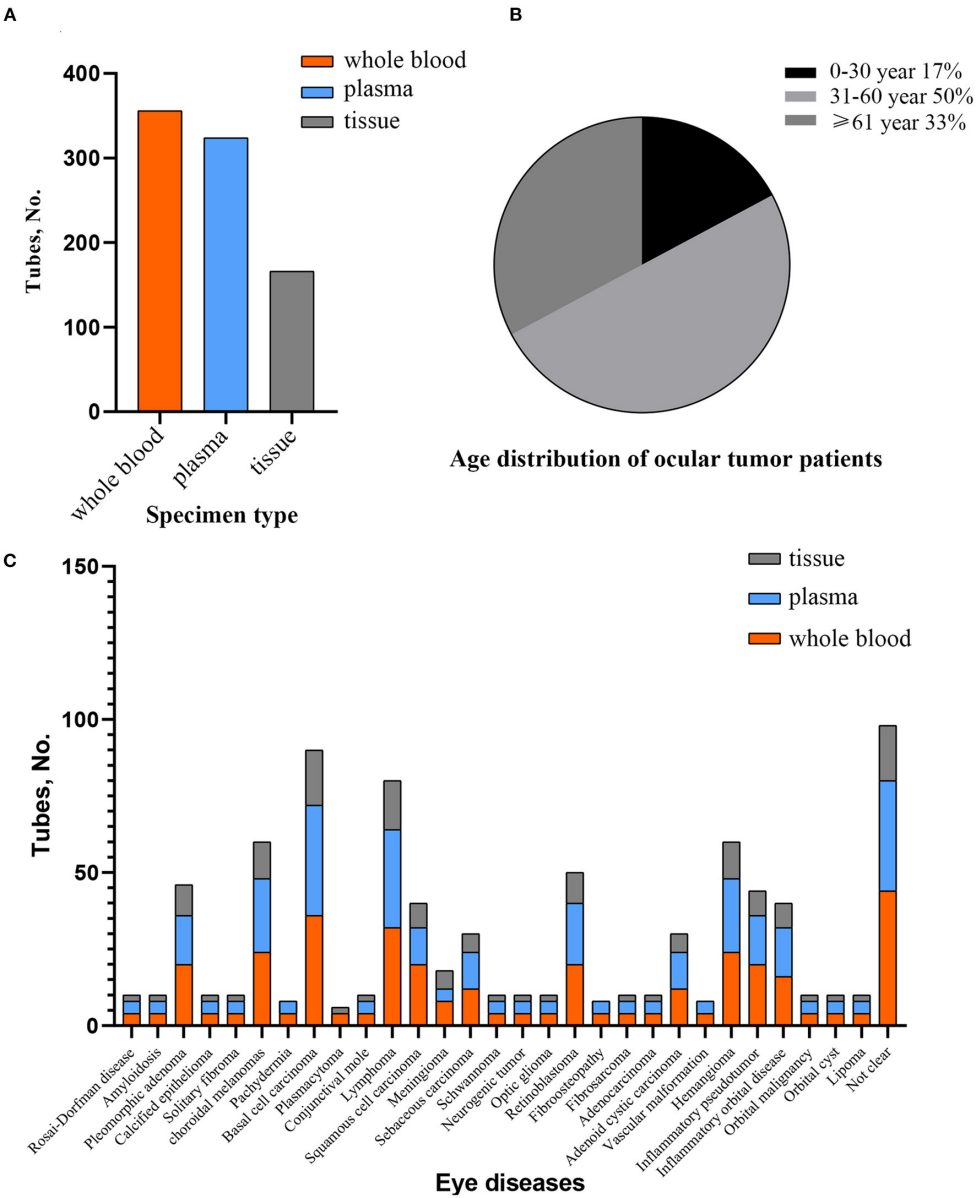
The overview of specimen collection. **(A)** Number of samples according to different types in preservation. **(B)** Age distribution of eye tumor cases. **(C)** Specimen's condition concerning various kinds of suspected or diagnosed ocular tumor stored in biobank.

The total number of samples reached 846, including 356 blood samples, 324 plasma samples, and 166 tissue samples, which accounted for 42.1, 38.3, 19.6% of all the samples, respectively (Figure 2). All samples were stored in a −80°C freezer. Age distribution of ocular tumor patients showed the highest representation for an age group 31–60 years old. From the total number of cases, the interval of age between 0 and 30 accounted for 17%, and the interval of age between 31 and 60 accounted for 50%, and patients older than 60 accounted for 33% (Figure 2). Currently, the biobank preserves biological samples from 92 subjects, which covered 21 types of eye tumors and several eye diseases. These included pleomorphic adenoma (5%), choroidal melanoma (7%), basal cell carcinoma (10%), lymphoma (9%), squamous cell carcinoma (5%), meningioma (3%), sebaceous carcinoma (3%), retinoblastoma (5%), adenoid cystic carcinoma (3%), hemangioma (7%), and other ocular tumors that accounted for <1% (Figure 2 and Table 1).

**Table 1:** Overview of specimen collection of ocular tumor biobank.

**Eye diseases**	**Subject (%) *n* = 92**	**Whole blood, No**.	**Plasma, No**.	**Tissue No**.	**Total No. (%)**	**Storage condition**
Rosai-Dorfman disease	1 (1)	4	4	2	10 (1.2)	−80°C
Amyloidosis	1 (1)	4	4	2	10 (1.2)	−80°C
Pleomorphic adenoma	5 (5)	20	16	10	46 (5.4)	−80°C
Calcified epithelioma	1 (1)	4	4	2	10 (1.2)	−80°C
Solitary fibroma	1 (1)	4	4	2	10 (1.2)	−80°C
choroidal melanomas	6 (7)	24	24	12	60 (7.1)	−80°C
Pachydermia	1 (1)	4	4	0	8 (0.9)	−80°C
Basal cell carcinoma	9 (10)	36	36	18	90 (10.6)	−80°C
Plasmacytoma	1 (1)	4	0	2	6 (0.7)	−80°C
Conjunctival mole	1 (1)	4	4	2	10 (1.2)	−80°C
Lymphoma	8 (9)	32	32	16	80 (9.5)	−80°C
Squamous cell carcinoma	5 (5)	20	12	8	40 (4.7)	−80°C
Meningioma	3 (3)	8	4	6	18 (2.1)	−80°C
Sebaceous carcinoma	3 (3)	12	12	6	30 (3.5)	−80°C
Schwannoma	1 (1)	4	4	2	10 (1.2)	−80°C
Neurogenic tumor	1 (1)	4	4	2	10 (1.2)	−80°C
Optic glioma	1 (1)	4	4	2	10 (1.2)	−80°C
Retinoblastoma	5 (5)	20	20	10	50 (5.9)	−80°C
Fibro osteopathy	1 (1)	4	4	0	8 (0.9)	−80°C
Fibrosarcoma	1 (1)	4	4	2	10 (1.2)	−80°C
Adenocarcinoma	1 (1)	4	4	2	10 (1.2)	−80°C
Adenoid cystic carcinoma	3 (3)	12	12	6	30 (3.5)	−80°C
Vascular malformation	1 (1)	4	4	0	8 (0.9)	−80°C
Hemangioma	6 (7)	24	24	12	60 (7.1)	−80°C
Inflammatory pseudotumor	5 (5)	20	16	8	44 (5.2)	−80°C
Inflammatory orbital disease	4 (4)	16	16	8	40 (4.7)	−80°C
Orbital malignancy	1 (1)	4	4	2	10 (1.2)	−80°C
Orbital cyst	1 (1)	4	4	2	10 (1.2)	−80°C
Lipoma	1 (1)	4	4	2	10 (1.2)	−80°C
Not clear	13 (14)	44	36	18	98 (11.6)	−80°C

### Demographic Information

The average age of patients with benign tumors was 41.5 years, ranging from 1 to 65 years (Table 2). In contrast, that of patients with malignant tumors was 50.9 years, with a range from 10 months to 80 years. Among all the male participants, 12.5% of patients were diagnosed with benign tumors compared with 87.5% of patients with malignant tumors. For female participants, the prevalence of patients with benign and malignant tumors was comparable. More than 98% of participants belonged to the Han ethnic group. From all the donors' residence information recorded, the incidence of patients with malignant tumors was 68% in the city, compared to 70% in rural areas. Also, tumors were more likely to occur in one eye instead of both eyes (95 vs. 5%). A total of 56% of ocular tumors occurred in the left eye compared with 39% in the right eye (Table 2).

**Table 2:** Demographics of donors with ocular tumors.

**Variables**	**Benign ocular tumor**	**Malignant ocular tumor**
	**(*n* = 20), No. (%)**	**(*n* = 44), No. (%)**
**Age at presentation, years**		
Mean	41.5	50.9
Range (minimum-maximum)	1–65	0.8–80
**Gender**		
Male	4 (12.5)	28 (87.5)
Female	16 (50)	16 (50)
**Ethnicity**		
Han	20 (32)	43 (68)
Others	0 (0)	1 (100)
**Residence**		
City	13 (32)	28 (68)
Rural area	7 (30)	16 (70)
**Diseased eye**		
Left	13 (36)	23 (64)
Right	6 (24)	19 (76)
Bilateral	1 (33)	2 (67)

### Clinical Characteristics

Clinical features are listed in Table 3. Malignant ocular tumors were more common in males than in females (88 vs. 50%; *P* = 0.001). We compared the prevalence of malignant ocular tumors between patients under 40 and older than 40 years old. Malignant tumors tended to occur in older patients than younger patients (76 vs. 50%; *P* = 0.043). The occurrence of the various presenting complaints, including eyelid swelling, ulceration, vision diminution, eye watering, leukocoria, strabismus, occluding sensation, and pain was comparable in both the benign and malignant ocular tumors groups. There was a higher occurrence of a lump in participants with malignant ocular tumors than in benign tumor' participants (59 vs. 30%; *P* = 0.031). In comparison, the symptom of proptosis was more likely to occur in patients with benign tumors than in patients with malignant tumors (55 vs. 7%; *P* < 0.001). The condition of whether the tumor was palpable was analyzed among all the participants. It was more common for tumors to be palpable in patients with malignant ocular tumors than in patients with benign tumors (71 vs. 30%; *P* = 0.002). Among patients with benign tumors, 2 occurred on the ocular surface, 14 occurred in orbital and adnexal lesions, and none occurred intraocularly. For patients with malignant ocular tumors, there were 18 (90%), 16 (53%), and 9 (100%) cases with tumors on the ocular surface, orbital and adnexal lesions as well as intraocularly, respectively. The prevalence of the malignant ocular tumors of the ocular surface and intraocular was higher than that of orbital and adnexal lesions (*P* = 0.003). Factors predictive of malignant ocular tumors are listed in Table 4. Using multivariate analysis, factors predictive of malignant ocular tumors included gender (B = 1.599; *P* = 0.025). The symptom of proptosis (B = −2.534; *P* = 0.001) was the protective factor for malignant ocular tumors.

**Table 3:** Clinical and histological features of patients with ocular tumors.

**Variables**	**Benign ocular tumor**	**Malignant ocular tumor**	***P***
	**(*n* = 20), No. (%)**	**(*n* = 44), No. (%)**	
**Age**			
<40	9 (50)	9 (50)	0.043
≥40	11 (24)	35 (76)	
**Gender**			
Male	4 (13)	28 (88)	0.001
Female	6 (50)	16 (50)	
**Diseased eye**			
Left	13 (36)	23 (64)	0.660
Right	6 (24)	19 (76)	
Bilateral	1 (33)	2 (67)	
**Presenting complaints**			
Eyelid swelling	3 (15)	4 (9)	0.668
Ulceration	0 (0)	2 (5)	1.000
Lump	6 (30)	26 (59)	0.031
Proptosis	11 (55)	3 (7)	0
Vision diminution	2 (10)	4 (9)	1
Eye watering	1 (10)	1 (2)	0.531
Leukocoria	0	4 (9)	0.30
Strabismus	0	1 (2)	1
Occluding sensation	0	1 (2)	1
Pain	0	1 (2)	1
**Categorical division of duration of symptoms (*****n*** **=** **64), months**			
≤12	10 (50)	28 (64)	0.303
>12	10 (50)	16 (36)	
No results	1 (5)	6 (14)	
**Intraocular pressure, mmHg**			
Normal	18 (90)	37 (84)	1
High	2 (10)	4 (9)	
No results	0	3 (7)	
**Tumor size**			
Cannot be touched	14 (70)	13 (30)	0.002
Can be touched	6 (30)	31 (71)	
**Lymph node involvement**			
Yes	0 (0)	12 (27)	
No	0 (0)	5 (11)	
Not clear	20 (100)	27 (61)	
**Whether has cancer history**			
Yes	3 (15)	3 (7)	0.359
No	16 (80)	40 (91)	
Not clear	1 (5)	1 (2)	
**Eye movements**			
Limited	9 (45)	10 (23)	0.08
Not limited	10 (45)	28 (64)	
**Histological Features**			
**Location of eye tumors**			
Ocular surface	2 (10)	18 (90)	0.003
Intraocular	0	9 (100)	
Orbital and adnexal lesions	14 (46.7)	16 (53.3)	

**Table 4:** Variables of clinical features and demographics predicting the malignant ocular tumors by multivariate analysis.

**Variables**	**B**	**95% Exp (B)**	***P***
Gender	1.599	1.219–20.104	0.025
Proptosis	−2.534	0.017–0.371	0.001

## Discussion

### A Dedicated Ocular Tumor Biobank Containing Biological Specimens and Associated Clinical Data

With the rapid growth of knowledge and innovations, biomedical research and healthcare are shifting toward the vision of personalized and translational medicine (15). In this field, high-quality and well-annotated biospecimens are essential. Biobanks facilitate the transformation of basic medical research into clinical research studies by promoting the development of drugs, the discovery of biomarkers, and the optimization of therapies. Meanwhile, biobanks accelerate human research, with less reliance on animal models and advancing research based on human biospecimen resources.

Biobanks have been previously established for several tumors, including prostate (16), breast (17), gastric (18), glioblastoma (19), lung (20), and ovarian cancer (21), which provided valuable resources for translational and molecular studies. Given the lack of ocular tumor biobanks in China, we established an ocular tumor biobank to provide a resource from different patients covering 21 kinds of eye tumors, including blood, plasma, and tissue samples (Figure 2 and Table 1). Associated data include patient characteristics, demographics, oncological diagnosis, clinical details (e.g., symptoms, duration time, examination results, and family history) as well as histological features (Tables 2, 3). Clinical data is of equal significance with biological information contained in a biospecimen. Combining both can facilitate comprehensive research on epidemiologic, etiology, and molecular mechanisms of eye tumors. We also report developing and operating the eye tumor biobank, including biobank design, collection, transportation, processing, and preservation of biological samples and collection of clinical data, aiming to provide guidance and experience for researchers interested in biobanking work (Figure 1).

### Strengths

Currently, our ocular tumor biobank is established and has been in operation for over 1 year. It provides opportunities for researchers to study ocular tumors at the molecular and clinical levels, facilitating a comprehensive understanding of various ocular tumors. Four of the main benefits and potential uses of our eye tumor biobank initiative are worthy of note. First, we made a concerted effort to establish the ocular tumor biobank based on the cooperation between an academic agency and a hospital. Hospitals have a variety and quantity of biological sample resources but lack dedicated specimen management. This leads to an uncertainty of specimen quality which limits the use of samples. On the other hand, an academic agency can manage biological samples and has the expertise to use samples to perform scientific research. However, the shortage of sample resources hampers their progress. Our biobank can achieve complementary advantages by promoting collaboration between academic agencies and hospitals. It can improve eye tumor research developments and help clinicians obtain financial support from projects, further supporting the biobank's sustainable development. Second, unlike other biobanks that provide biological samples and limited clinical data (22), our biobank features various sample resources along with plentiful associated epidemiological and clinical data, which is critical for improving the comprehensive understanding of various kinds of eye tumors and defining new biomarkers for the diagnosis of patients with ocular tumors. Our biobank provides the opportunity to make correlations and analyze the relationship between biological information and other data, including epidemiological and social data, various clinical situations, and follow-up, helping identify risk or protective factors of eye tumors, thereby improving the diagnostic decisions of clinicians. Third, the biobank contains specimens covering 21 types of ocular tumors and does not focus solely on the major tumor types. This makes it available for supporting various studies of different kinds of eye tumors. Furthermore, our biobank can provide valuable human-derived sample resources for research on eye tumors that lack of adequate cell lines or a suitable animal model which can mimic the structures and functional properties of ocular tumors (9, 23). Lastly, our biobank has standardized procedures and specialized personnel for management of samples as well as data. This approach can help with providing samples and data of high-quality which can promote research into tumor heterogeneity, identifying new prognostic factors and developing novel therapies, and help avoid variability in sample quality during the collection, transportation, processing, and preservation of samples.

### Limitations

While a tremendous effort has been made in setting up the ocular tumor biobank, limitations exist at the present and may persist for a certain time. Thus, insufficient numbers of samples for each tumor type limited the contributions of our biobank to ocular tumor research. There were several reasons. First, our biobank has been in operation for only 14 months. We have been in the initial stages of exploring how to develop an ocular tumor biobank and accumulating experience in the smooth operation of a biobank. Second, we only collaborated with one hospital, where the number of patients was limited. Lastly, even though clinicians explained the concept of biobanks to patients, patients lacked a full understanding of the purpose of biobanks, which hindered their sample donations. Although we have a limited number of cases for each tumor, we covered nearly 21 types of ocular tumor including some rare tumors, which can provide material for ocular tumor research. Moreover, we identified solutions to solve various problems. First, we plan to cooperate with ophthalmology departments of the other affiliated hospitals that belong to our school, to recruit more donors in developing the ocular tumor biobank. More samples and associated data will be collected in a shorter time with more participation. On the other hand, valuable experience has been obtained and biobankers have been trained during the past year of operating the biobank, which can greatly improve efficiency in collecting and processing samples and can contribute to improved governance of the ocular tumor biobank. Another challenge was the preservation condition of samples in our biobank. The WHO-IARC document described the minimum technical standards and protocols for biological resource centers and recommends that tissue samples should be stored at below −130°C (24). Brockbank (25) demonstrated that storage temperatures below −135°C are necessary for retention of protein synthetic functions. Most of the samples were preserved in the freezers at −80°C owing to the lack of enough liquid nitrogen containers in our biobank. However, there is a study showing that the samples' quality did not significantly change under different conditions (−80°C, −196°C, and RNAlater/−80°C) (26).

### Sustainable Development

It is difficult to maintain the biobank's sustainable development. Lack of financial support is one of the common factors that hampers biobanks' development. Many biobanks in Europe and the United States rely on public agency funding, university institutional funding, or external funding, and the funds can sometimes be insufficient. The ocular tumor biobank obtained financial support mainly from the national project in the early stages, and we expect to meet basic running costs through the following ways in the future. On the one hand, we can help physicians with applying for project support from the national and other funding institutions. We receive funding support in return as the project coordinator. On the other hand, we could obtain financial support by providing several experimental services or biobank related training courses for people who do not have their own laboratory or who want to construct their own biobank.

### Future Development

We will continue to accumulate samples together with epidemiological and clinical information, to build a more sizable biobank that can provide opportunities to support population-based studies as well as genetic studies. Meanwhile, improving cooperation between the biobank and ophthalmology department of many other hospitals, to develop an ocular tumor biobank network, is our next goal, which can help provide plentiful specimen and data resources quickly for researchers. Furthermore, patient-derived tumor xenografts and tumor organoids have become significant preclinical models for cancer research. Both models maintain critical features from their parental tumors, such as genetic and phenotypic heterogeneity, which allows them to study a broad spectrum of applications. To further the potential of the ocular tumor biobank, we will explore methods for developing xenografts and organoids of different types of ocular tumors using human-derived tumor resources.

The ocular tumor biobank provides access to a large repository of blood and tissue samples and epidemical and clinical data for researchers. Our biobank contributes to accelerating progress in the pathogenesis research and clinical use of biomarkers for the diagnosis and prognosis of ocular tumors, aiming to promote precision and personalized medicine in the future.

## Data Availability Statement

The raw data supporting the conclusions of this article will be made available by the authors, without undue reservation.

## Ethics Statement

The studies involving human participants were reviewed and approved by the institutional review board (IRB) of the Third Xiangya Hospital of Central South University. Written informed consent to participate in this study was provided by the participant, or the participants' legal guardian/next of kin.

## Author Contributions

JH, HY, and JT developed the ocular tumor biobank. All authors contributed to the study concept and design. ZG, ZZ, YZ, HY, and MZ contributed to the processing, transportation and storage of biological samples, and data acquisition. JT and SW enrolled the patients with ocular tumor and collected samples at surgery. ZG performed the statistical analysis and wrote the manuscript. All authors contributed to the article and approved the submitted version.

## Funding

This work was funded by the National Key R&D Program of China (No. 2016YFC1201800), Key Research and Development Program of Hunan Province (No. 2018SK2090), the Fundamental Research Funds for the Central Universities of Central South University (No. 1053320192191), and the Research and Development Project of Central South University, China (No. 1053320213162).

## Conflict of Interest

The authors declare that the research was conducted in the absence of any commercial or financial relationships that could be construed as a potential conflict of interest.

## Publisher's Note

All claims expressed in this article are solely those of the authors and do not necessarily represent those of their affiliated organizations, or those of the publisher, the editors and the reviewers. Any product that may be evaluated in this article, or claim that may be made by its manufacturer, is not guaranteed or endorsed by the publisher.

## References

[1] Neupane R Gaudana R Boddu SHS. Imaging techniques in the diagnosis and management of ocular tumors: prospects and challenges. AAPS J. (2018) 20:97. 10.1208/s12248-018-0259-9 30187172

[2] Broaddus E Topham A Singh AD. Incidence of retinoblastoma in the USA: 1975-2004. Br J Ophthalmol. (2009) 93:21–3. 10.1136/bjo.2008.138750 18621794

[3] Seregard S Lundell G Svedberg H Kivela T. Incidence of retinoblastoma from 1958 to 1998 in Northern Europe: advantages of birth cohort analysis. Ophthalmology. (2004) 111:1228–32. 10.1016/j.ophtha.2003.10.023 15177976

[4] Kivela T. The epidemiological challenge of the most frequent eye cancer: retinoblastoma, an issue of birth and death. Br J Ophthalmol. (2009) 93:1129–31. 10.1136/bjo.2008.150292 19704035

[5] Singh AD Turell ME Topham AK. Uveal melanoma: trends in incidence, treatment, and survival. Ophthalmology. (2011) 118:1881–5. 10.1016/j.ophtha.2011.01.040 21704381

[6] Kujala E Makitie T Kivela T. Very long-term prognosis of patients with malignant uveal melanoma. Invest Ophthalmol Vis Sci. (2003) 44:4651–9. 10.1167/iovs.03-0538 14578381

[7] Singh AD Rennie IG Kivela T Seregard S Grossniklaus H. The Zimmerman-McLean-Foster hypothesis: 25 years later. Br J Ophthalmol. (2004) 88:962–7. 10.1136/bjo.2003.029058 15205248 PMC1772246

[8] Laurie N Mohan A McEvoy J Reed D Zhang J Schweers B. Changes in retinoblastoma cell adhesion associated with optic nerve invasion. Mol Cell Biol. (2009) 29:6268–82. 10.1128/MCB.00374-09 19786571 PMC2786692

[9] Susskind D Hurst J Rohrbach JM Schnichels S. Novel mouse model for primary uveal melanoma: a pilot study. Clin Exp Ophthalmol. (2017) 45:192–200. 10.1111/ceo.12814 27505446

[10] Benavente CA McEvoy JD Finkelstein D Wei L Kang G Wang YD. Cross-species genomic and epigenomic landscape of retinoblastoma. Oncotarget. (2013) 4:844–59. 10.18632/oncotarget.1051 23765217 PMC3757242

[11] Stamer WD Williams AM Pflugfelder S Coupland SE. Accessibility to and quality of human eye tissue for research: a cross-sectional survey of ARVO members. Invest Ophthalmol Vis Sci. (2018) 59:4783–92. 10.1167/iovs.18-25319 30304462

[12] Henderson GE Cadigan RJ Edwards TP Conlon I Nelson AG Evans JP. Characterizing biobank organizations in the U.S.: results from a national survey. Genome Med. (2013) 5:3. 10.1186/gm407 23351549 PMC3706795

[13] Best practices for repositories collection, storage, retrieval, and distribution of biological materials for research international society for biological and environmental repositories. Biopreserv Biobank. (2012) 10:79–161. 10.1089/bio.2012.1022 24844904

[14] Lang P Kim JW McGovern K Reid MW Subramanian K Murphree AL. Porous orbital implant after enucleation in retinoblastoma patients: indications and complications. Orbit. (2018) 37:438–43. 10.1080/01676830.2018.1440605 29461921 PMC6613564

[15] Le Tourneau C Kamal M Tredan O Delord JP Campone M Goncalves A. Designs and challenges for personalized medicine studies in oncology: focus on the SHIVA trial. Target Oncol. (2012) 7:253–65. 10.1007/s11523-012-0237-6 23161020

[16] Saifuddin SR Devlies W Santaolalla A Cahill F George G Enting D. King's Health Partners' Prostate Cancer Biobank (KHP PCaBB). BMC Cancer. (2017) 17:784. 10.1186/s12885-017-3773-8 29166865 PMC5700705

[17] Braicu C Berindan-Neagoe I Pileczki V Cojocneanu-Petric R Pop LA Puscas E. Breast tumor bank: an important resource for developing translational cancer research in Romania. Cancer Biomark. (2014) 14:119–27. 10.3233/CBM-130309 24878812 PMC12928375

[18] Mariette C Renaud F Piessen G Gele P Copin MC Leteurtre E. The FREGAT biobank: a clinico-biological database dedicated to esophageal and gastric cancers. BMC Cancer. (2018) 18:139. 10.1186/s12885-018-3991-8 29409462 PMC5801889

[19] Clavreul A Soulard G Lemee JM Rigot M Fabbro-Peray P Bauchet L. The French glioblastoma biobank (FGB): a national clinicobiological database. J Transl Med. (2019) 17:133. 10.1186/s12967-019-1859-6 31014363 PMC6480741

[20] Yu K Zhang J Li X Xu L Zhang Y Xing J. Establishment and management of a lung cancer biobank in Eastern China. Thorac Cancer. (2015) 6:58–63. 10.1111/1759-7714.12144 26273336 PMC4448472

[21] De Gregorio A Nagel G Moller P Rempen A Schlicht E Fritz S. Feasibility of a large multi-center translational research project for newly diagnosed breast and ovarian cancer patients with affiliated biobank: the BRandO biology and outcome (BiO)-project. Arch Gynecol Obstet. (2020) 301:273–81. 10.1007/s00404-019-05395-3 31781887

[22] Skeie JM Tsang SH Zande RV Fickbohm MM Shah SS Vallone JG. A biorepository for ophthalmic surgical specimens. Proteomics Clin Appl. (2014) 8:209–17. 10.1002/prca.201300043 24115637 PMC3964151

[23] Tasiou V Hiber M Steenpass L. A Mouse Model for Imprinting of the Human Retinoblastoma Gene. PLoS ONE. (2015) 10:e0134672. 10.1371/journal.pone.0134672 26275142 PMC4537222

[24] Mendy M Caboux E Lawlor RT Wright J Wild CP. Common Minimum Technical Standards and Protocols for Biobanks Dedicated to Cancer Research. Lyon: IARC Technical Publications (2017). 33539055

[25] Brockbank KG Carpenter JF Dawson PE. Effects of storage temperature on viable bioprosthetic heart valves. Cryobiology. (1992) 29:537–42. 10.1016/0011-2240(92)90058-A 1424711

[26] Zhang G Xia B Liu T Zhang J Niu M Xu S. A high-quality biobank supports breast cancer research in Harbin, China. Biopreserv Biobank. (2016) 14:375–82. 10.1089/bio.2015.0010 27082785

